# Tissue-invasive CMV disease is associated with poor prognosis during remission induction therapy for autoimmune inflammatory rheumatic diseases

**DOI:** 10.3389/fimmu.2026.1696516

**Published:** 2026-02-12

**Authors:** Hideki Oka, Shuji Sumitomo, Chisato Miyakoshi, Koichiro Ohmura

**Affiliations:** 1 Department of Rheumatology, Kobe City Medical Center General Hospital, Kobe, Hyogo, Japan; 2 Department of Research Support, Center for Clinical Research and Innovation, Kobe City Medical Center General Hospital, Kobe, Hyogo, Japan

**Keywords:** connective tissue diseases, cytomegalovirus, cytomegalovirus infection, cytomegalovirus disease, prognosis

## Abstract

**Objectives:**

Cytomegalovirus (CMV) is a notable health concern in immunocompromised individuals and presents as CMV reactivation (CMV infection) or CMV reactivation with tissue invasion (CMV disease). However, few studies have distinguished these CMV patterns during induction therapy for autoimmune inflammatory rheumatic diseases (AIIRDs). This study investigated the clinical characteristics of patients with CMV disease, diagnosed through histopathology, immunohistochemistry, or polymerase chain reaction, during induction therapy for AIIRDs in comparison with those of patients with CMV infection.

**Methods:**

This single-center retrospective study included patients with AIIRDs undergoing remission induction therapy with glucocorticoids ≥20 mg/day for ≥4 weeks, hospitalized between 2013 and 2024. Data on clinical characteristics, treatments, and outcomes were collected, and CMV disease, determined by histopathological findings, was compared with CMV infection.

**Results:**

Among 82 patients administered AIIRDs and positive for CMV antigenemia, 14 had CMV disease and 68 had CMV infection. CMV disease was associated with lower platelet counts, higher aspartate aminotransferase/alanine transaminase levels, and higher maximum antigenemia. The prevalence of coincident infections (93% vs. 32%) and mortality rate (57% vs. 24%) were higher in patients with CMV disease. Kaplan–Meier analysis showed poorer survival for patients with CMV disease (log-rank test, p=0.027). Receiver operating characteristic analysis identified antigen count (cutoff: 36) as the best predictor of CMV disease.

**Conclusion:**

CMV disease during remission induction therapy for AIIRDs is associated with higher antigenemia, more coincident infections, and poorer survival compared with CMV infection. Identifying the risk factors for CMV disease, including antigenemia and coincident infections, can improve patient outcomes.

## Introduction

Cytomegalovirus (CMV) is a double-stranded DNA virus belonging to the family Herpesviridae. After primary infection, CMV enters a latent state and can reactivate, especially among immunocompromised individuals (1, 2). CMV reactivation can cause constitutional symptoms such as fever and fatigue, as well as organ damage in the digestive tract, lungs, retina, liver, and central nervous system (3).

CMV infection is defined as the isolation of the virus or detection of viral proteins or nucleic acid in the blood or other specimens (4). In contrast, CMV disease is defined by the evidence of CMV infection in the body tissues accompanied by clinical symptoms (4). CMV infection precedes CMV disease, with risk factors including hypoalbuminemia and CMV pp65 antigen levels >5.6/10^5^ (5), although not all CMV infection cases progress to CMV disease. Reactivation of the virus is associated with a high mortality rate during remission induction therapy for autoimmune inflammatory rheumatic diseases (AIIRDs) (6).

The definition of CMV disease during AIIRD in various observational remains inconsistent (5–8). The gold standard for the diagnosis of CMV disease is the identification of CMV infection in the affected organs along with clinical symptoms (4, 9). However, owing to the challenges of tissue diagnosis, a combination of clinical symptoms related to the affected organs and evidence of CMV reactivation is often used as an alternative diagnostic approach. During remission induction therapy, CMV disease is not easily distinguishable from drug-induced effects and underlying disease activity, which can manifest as decreased platelet count, liver dysfunction, and gastrointestinal symptoms.

This study aimed to investigate the clinical characteristics of patients with CMV disease, diagnosed via histopathology, immunohistochemistry, or polymerase chain reaction (PCR), in cases of CMV reactivation during induction therapy for AIIRDs in comparison to those of patients with CMV infection. Through this study, more accurate characteristics of CMV disease during remission induction therapy, a critical aspect of CMV management, can be identified.

## Methods

### Patients

This single-center retrospective observational study investigated the clinical characteristics of CMV infection and CMV disease during induction therapy for AIIRDs. Patients aged ≥18 years who were hospitalized at the Department of Rheumatology and General Internal Medicine at Kobe City Medical Center General Hospital between January 2013 and March 2024, received remission induction therapy for AIIRDs, and underwent CMV antigenemia testing were included. Remission induction treatment was defined as the administration of glucocorticoids at a dose ≥20 mg/day for ≥4 weeks, regardless of the concomitant use of immunosuppressants or biologics. Patients who were CMV pp65 antigen-positive before initiating treatment were excluded.

This study was approved by the Ethics Committee of Kobe City Medical Center General Hospital (zn231011). In accordance with Japanese regulations, the need for written informed consent from patients was waived. The study details were published on the hospital’s website, providing patients with an opportunity to opt out. This study was conducted in accordance with the principles of the Declaration of Helsinki.

### Data collection

Data on underlying diseases, age, sex, disease duration, affected organs, treatment drugs, blood tests when CMV pp65 antigen was positive, treatment initiation date, number of days from treatment initiation to the first CMV pp65 antigen positivity, antigen count and glucocorticoid dose at that time, maximum CMV pp65 antigen count and glucocorticoid dose at that time, organs affected by CMV, and all-cause mortality, were retrieved from the medical records.

### CMV pp65 antigenemia assay and antiviral therapy

The CMV pp65 antigenemia assay for detecting CMV antigen-positive cells (polymorphonuclear leukocytes) in peripheral blood was performed using the C10/C11 method (LSI Medience Corporation, Japan), an indirect enzyme antibody technique employing a monoclonal antibody against the CMV pp65 antigen. The number of pp65 antigen-positive cells out of 5.0×10^4^ polymorphonuclear leukocytes per slide was counted, with the total count from two slides (1.0×10^5^ cells) used in this study. The timing of the CMV pp65 antigen test was at the discretion of the attending physician. Generally, it was performed within 3 months of initiating remission induction therapy, and patients who tested positive were included in the study. If the underlying disease recurred, and the same patient experienced multiple episodes of CMV pp65 antigen positivity, only the first positive episode was considered.

Antiviral therapy was administered to all patients diagnosed with tissue-invasive CMV disease. For patients with CMV antigenemia without tissue invasion, preemptive antiviral therapy was typically considered when the pp65 antigenemia count reached ≥10 positive cells per 1.0×10^5 polymorphonuclear leukocytes, or at the discretion of the treating physician based on the overall clinical context. Ganciclovir was used as the first-line agent, and foscarnet was used when ganciclovir was contraindicated or not tolerated.

### Definition of CMV infection, disease, and coincident infection

CMV infection was defined as CMV reactivation, indicated by at least one positively stained polymorphonuclear leukocyte in the CMV pp65 antigenemia assay, without documentation of CMV in the affected tissues.

CMV disease was defined as the presence of clinical symptoms and/or signs of organ involvement, along with the documentation of CMV in the affected tissues (4, 9). In this study, the organs affected were the gastrointestinal tract, lungs, and eyes. Gastrointestinal CMV disease was determined by the detection of CMV-positive cells in tissue specimens through immunohistochemical staining, in addition to macroscopic mucosal lesions, such as redness, erosion, and ulcers on upper or lower gastrointestinal endoscopy. CMV pneumonitis was indicated by abnormal shadows on chest imaging, with CMV-positive cells detected in tissue specimens from the lung (e.g., lung biopsy tissue) by immunohistochemical staining or detection of CMV DNA in bronchoalveolar lavage fluid by PCR. CMV retinitis was identified based on the characteristic retinal findings observed during ophthalmologic examination.

Coincident infections were defined as infections requiring initiation of antibacterial and/or antifungal therapy that were diagnosed on or after the date of first CMV pp65 antigenemia positivity (index date) and occurred during the CMV antigenemia-positive period. Infections requiring antimicrobial therapy that occurred before the index date were also recorded and are presented separately in **Supplementary Table 1**. Severe infection was defined as an infection that required intensive care unit admission and/or vasopressor support. All-cause mortality, regardless of its relation to CMV infection, was recorded for each patient.

### Statistical analysis

Comparisons between patients with CMV disease and those with CMV infection were performed using Pearson’s chi-square test for large sample sizes and Fisher’s exact test for small expected frequencies of categorical variables. Wilcoxon rank-sum test was used for comparison of continuous variables. Categorical variables are expressed as frequencies and percentages and continuous variables as median and 25th to 75th percentiles. To identify factors associated with CMV disease, logistic regression analysis was performed using platelet count, alanine transaminase (ALT) level, maximum CMV antigenemia, and coincident infections. Variables were selected *a priori* based on clinical plausibility and univariable between-group differences, while limiting the number of covariates to reduce overfitting given the small number of CMV disease events. AST was not included together with ALT because of collinearity between transaminases; ALT was chosen as the representative marker of hepatocellular injury. Overall survival was analyzed using the Kaplan–Meier method and the log-rank test. Cox proportional hazards models were used to determine the hazard ratios. To determine the optimal cutoff values for CMV antigenemia, platelet count, and ALT levels for predicting CMV disease, receiver operating characteristic (ROC) curves were constructed, with Youden’s index used to maximize sensitivity and specificity.

All analyses were two-sided, and the significance level was set at p < 0.05. All statistical analyses were performed using R version 4.3.1 (The R Foundation for Statistical Computing, Vienna, Austria).

## Results

### Baseline characteristics

Among 518 patients who received remission induction therapy during hospitalization, 271 (52%) underwent the CMV pp65 antigenemia assay, while 247 (48%) did not. Among the patients who underwent the assay, 82 (30%) developed CMV pp65 antigenemia positivity within 3 months after initiation of induction therapy, whereas 189 remained CMV pp65 antigenemia-negative during the same period. Among these patients, 14 had CMV disease and 68 had CMV infection. The baseline characteristics of the participants are presented in **Table 1**. Overall, 68% of the patients were female, and the median age at the start of remission induction therapy was 74 years. Ninety-five percent of the patients were receiving their first remission induction therapy. The median follow-up period from the start of remission induction therapy to the end of the study was 461 days. The underlying AIIRDs included vasculitides in 52% of the patients, systemic lupus erythematosus in 15%, polymyositis/dermatomyositis in 12%, and Still’s disease in 12%. The specific types of vasculitides were giant cell arteritis (n=3), Takayasu arteritis (n=1), immunoglobulin A vasculitis (n=3), polyarteritis nodosa (n=2), eosinophilic granulomatosis with polyangiitis (n=2), and anti-neutrophil cytoplasmic antibody-associated vasculitis (n=32). The two groups showed no significant difference in terms of organ involvement. In the treatment of the underlying AIIRDs, glucocorticoid pulse therapy was performed in 67% of all cases, with a median maximum glucocorticoid dose of 50 mg/day. The two groups showed no difference in the use of concomitant immunosuppressants and biological agents.

**Table 1:** Baseline characteristics, treatment, and laboratory of CMV infection and CMV disease.

Variable	Overall (N = 82)^a^	CMV infection (N = 68)^a^	CMV disease (N = 14)^a^	p-value^b^
Female	56/82 (68%)	47/68 (69%)	9/14 (64%)	0.758
Age at diagnosis	74 (61, 81)	73 (60, 79)	76 (65, 83)	0.318
New onset	78/82 (95%)	64/68 (94%)	14/14 (100%)	1.000
Follow-up period, days	461 (175, 875)	476 (245, 860)	232 (77, 1,093)	0.550
Diagnosis				
Vasculitides	43/82 (52%)	36/68 (53%)	7/14 (50%)	1.000
SLE	12/82 (15%)	10/68 (15%)	2/14 (14%)	1.000
PM/DM	10/82 (12%)	8/68 (12%)	2/14 (14%)	0.677
Still’s disease	10/82 (12%)	8/68 (12%)	2/14 (14%)	0.677
Others	7/82 (8.5%)	6/68 (8.8%)	1/14 (7.1%)	1.000
Organ involvement				
Lung	48/82 (59%)	41/68 (60%)	7/14 (50%)	0.679
Heart	4/82 (4.9%)	4/68 (5.9%)	0/14 (0%)	1.000
Kidney	39/82 (48%)	33/68 (49%)	6/14 (43%)	0.926
Nerve	11/82 (13%)	9/68 (13%)	2/14 (14%)	1.000
Skin	31/82 (38%)	26/68 (38%)	5/14 (36%)	1.000
ENT/Eye	11/82 (13%)	10/68 (15%)	1/14 (7.1%)	0.680
Gastrointestinal tract	14/82 (17%)	10/68 (15%)	4/14 (29%)	0.245
Muscle/Joint	22/82 (27%)	19/68 (28%)	3/14 (21%)	0.749
Blood	14/82 (17%)	10/68 (15%)	4/14 (29%)	0.245
Large vessel	3/82 (3.7%)	3/68 (4.4%)	0/14 (0%)	1.000
Treatment				
Maximum GCs, mg	50 (45, 60)	55 (45, 60)	50 (46, 60)	0.708
Steroid pulse	55/82 (67%)	43/68 (63%)	12/14 (86%)	0.128
RTX	25/82 (30%)	19/68 (28%)	6/14 (43%)	0.341
CYC	17/82 (21%)	15/68 (22%)	2/14 (14%)	0.723
TNFi	2/82 (2.4%)	2/68 (2.9%)	0/14 (0%)	1.000
CNI	18/82 (22%)	13/68 (19%)	5/14 (36%)	0.177
MMF	3/82 (3.7%)	2/68 (2.9%)	1/14 (7.1%)	0.434
MTX	2/82 (2.4%)	1/68 (1.5%)	1/14 (7.1%)	0.318
AZA	2/82 (2.4%)	2/68 (2.9%)	0/14 (0%)	1.000
bDMARD	9/82 (11%)	7/68 (10%)	2/14 (14%)	0.647
Laboratory				
Alb, g/dL	2.60 (2.20, 3.00)	2.65 (2.28, 3.10)	2.30 (2.20, 2.60)	0.062
WBC,/µL	8,250 (6,325, 11,775)	8,450 (6,650, 12,300)	6,500 (5,775, 8,600)	0.062
Neutrophil, %	7,132 (5,126, 10,403)	7,508 (5,486, 10,726)	5,841 (4,668, 8,156)	0.113
Lymphocyte, %	442 (202, 800)	496 (209, 822)	272 (158, 566)	0.119
Hemoglobin, g/dL	10.00 (8.73, 11.10)	10.00 (8.78, 11.10)	10.45 (8.83, 11.28)	0.824
Platelet, ×10^4/^µL	180,000 (99,250, 234,000)	185,500 (110,750, 248,250)	98,000 (60,250, 170,250)	**0.013**
AST, U/L	20 (15, 33)	19 (15, 31)	43 (21, 60)	**0.006**
ALT, U/L	28 (16, 51)	24 (15, 43)	41 (33, 62)	**0.014**
Creatinine, mg/dL	0.76 (0.58, 1.65)	0.73 (0.59, 1.64)	0.94 (0.53, 2.54)	0.744
CRP, mg/dL	0.29 (0.07, 1.99)	0.24 (0.06, 1.98)	1.13 (0.21, 2.01)	0.244
Days to first Ag positive	21 (14, 31)	21 (13, 29)	27 (20, 53)	0.054
GC dose at first Ag positive, mg	45 (35, 54)	45 (30, 55)	40 (40, 50)	0.581
Number of maximum Ag	12 (3, 37)	8 (3, 24)	91 (39, 351)	**<0.001**
Death	24/82 (29%)	16/68 (24%)	8/14 (57%)	**0.021**
Antiviral therapy	47/82 (57%)	33/68 (49%)	14/14 (100%)	**<0.001**
Coincident infection	35/82 (43%)	22/68 (32%)	13/14 (93%)	**<0.001**

a n/N (%); Median (IQR, interquartile range).

b P-values were calculated using Fisher’s exact test, the Wilcoxon rank-sum test, and Pearson’s chi-squared test, with a value of <0.05 considered statistically significant for comparing CMV infection and CMV disease.

CMV, cytomegalovirus; SLE, systemic lupus erythematosus; PM, polymyositis; DM, dermatomyositis; ENT, Ear, Nose, and Throat; GCs, glucocorticoids; RTX, rituximab; CYC, cyclophosphamide; TNFi, tumor necrosis factor inhibitor; CNI, calcineurin inhibitor; MMF, mycophenolate mofetil; MTX, methotrexate; AZA, azathioprine; bDMARD, biological disease modifying anti-rheumatic drug; Alb, albumin (normal range of 3.9-4.9 g/dL); WBC, white blood cell (normal range of 3,900-9,800/µL); Neutrophil, normal range of 26-71%; Lymphocyte, normal range of 19-61%; Hemoglobin, normal range of 11.1-15.1 g/dL; Platelet, normal range of 13-37×10^4/^µL; AST, aspartate aminotransferase (normal range of 8–40 U/L); ALT, alanine aminotransferase (normal range of 8–40 U/L); Creatinine, normal range of 0.6-1.1 mg/dL; CRP, c-reactive protein (normal range of 0.0-0.5 mg/dL); Ag, antigenemia.

Bold values indicate p-values less than 0.005.

### Details of CMV disease and its risk factors

Among the 14 patients with CMV disease, the gastrointestinal tract was involved in 12, retina in one, and lungs in one. CMV antigenemia counts at first positivity varied by disease type: the median (IQR) count in gastrointestinal CMV disease (n=12) was 44.5 (5.75–294.5), whereas the single cases of CMV retinitis and CMV pneumonitis had counts of 250 and 1, respectively. Blood tests at the first CMV antigen positivity were compared between the CMV disease and CMV infection groups (**Table 1**). The median platelet count was significantly lower in the CMV disease group (98,000/µL) than in the CMV infection group (185,500/µL) (p=0.013). Aspartate aminotransferase and ALT levels were significantly higher in patients with CMV disease (p=0.006 and p=0.014, respectively). Although CMV disease tended to occur later than CMV infection, the difference in the time of first CMV antigen positivity after the start of remission induction therapy was not significantly different between the two groups (p=0.054). The median maximum CMV antigenemia count was 91 for CMV disease and 8 for CMV infection (p<0.001). The proportion of patients with CMV disease who had coincident infections during the period of positive CMV antigenemia was significantly higher than that of patients with CMV infection (93% vs. 32%, p<0.001). All patients with CMV disease received anti-CMV therapy. In contrast, 49% with CMV infection were treated with anti-CMV therapy (p<0.001). The proportion of all-cause death was higher in patients with CMV disease than in those with CMV infection (57% vs. 24%, p=0.021). The two groups showed no significant difference in the time from treatment initiation to the first positive result or in the dose of glucocorticoids at that time.

To identify factors associated with CMV disease, multivariable logistic regression analysis was performed using platelet count, ALT level, maximum CMV antigenemia count, and coincident infections (**Table 2**). Multivariable analysis showed that a higher maximum CMV antigenemia count (odds ratio (OR) 1.00, 95% confidence interval (CI) 1.00–1.01, p=0.01) and the presence of coincident infections (OR 20.2, 95% CI 2.74–493.47, p=0.014) were significantly associated with CMV disease. The most prevalent infections associated with CMV disease were oral and esophageal Candida infections (n=8). Among patients with CMV pp65 antigenemia positivity, the median time from initiation of remission induction therapy to the first CMV antigenemia positivity was 21 days, whereas the median time to the onset of coincident infection was 24 days. The types and timing of coincident infections relative to the first CMV antigenemia positivity are summarized in Supplementary **Supplementary Table 1**.

**Table 2:** Multivariable analysis of risk factors associated with CMV disease.

Variable	OR	95% CI	p-value
Platelet count	1.00	1.00–1.00	0.613
ALT	1.00	0.97–1.01	0.687
Maximum CMV Ag	1.00	1.00–1.01	**0.010**
Coincident infection	20.2	2.74–493.47	**0.014**

OR, Odds Ratio; CI, Confidence Interval; ALT, alanine aminotransferase; Ag, antigenemia; CMV, cytomegalovirus.

Bold values indicate p-values less than 0.005.

### Relationship between CMV disease and CMV antigenemia, platelet count, and ALT levels

**Figure 1:**
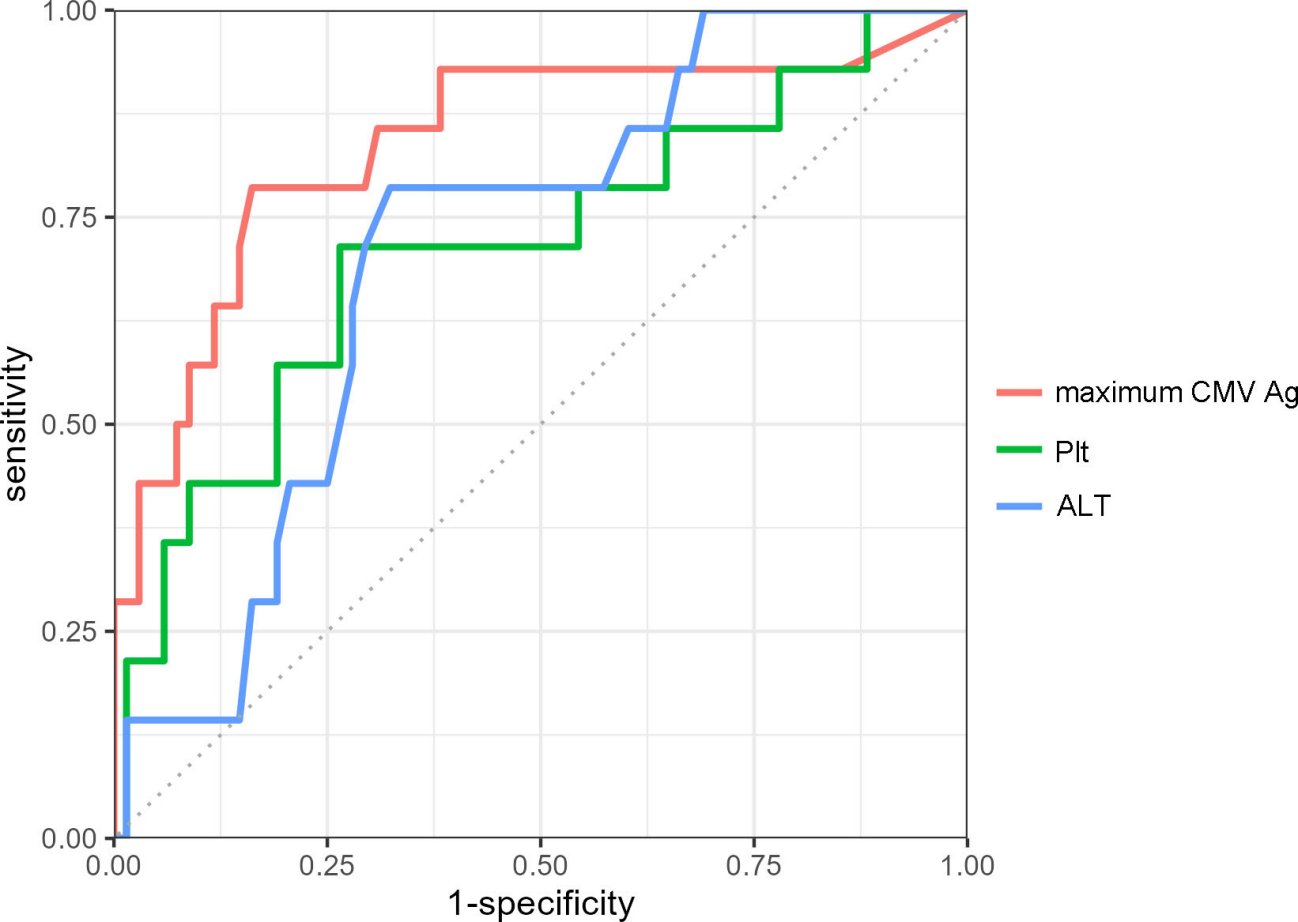
Receiver operating characteristic (ROC) curves for predicting CMV disease. The ROC curves depict the predictive ability of the maximum CMV antigen count (red), platelets (green), and ALT (blue) for CMV disease. The optimal cutoff value for CMV antigenemia was 36, with a sensitivity of 79% and specificity of 84% (area under the curve (AUC): 0.84). For platelet count and ALT level, the cutoff values were 115,500/µL (AUC: 0.71) and 33 U/L (AUC: 0.71), respectively. Among these variables, the maximum CMV antigen count demonstrated the highest predictive performance for CMV disease. ALT, alanine transaminase; CMV, Cytomegalovirus. Ag, antigenemia; Plt, platelet count.

The maximum CMV antigenemia count was associated with CMV disease in both univariate (**Table 1**) and multivariate (**Table 2**) analyses. The antigen count predicting CMV disease was 36 in the ROC curve, with a sensitivity of 79% and specificity of 84% (**Figure 1**). In the univariate analyses, platelets and ALT were also associated with CMV disease. The cutoff value for platelet count was 115,500/µL (sensitivity 71%, specificity 74%). For ALT, the cutoff value was 33 U/L (sensitivity 79%, specificity 68%). The areas under the curve (AUC) for maximum antigen count, platelet count, and ALT level were 0.84, 0.71, and 0.71, respectively.

### Prognosis of CMV disease and CMV infection

All-cause mortality in the CMV pp65 antigenemia assay population was 16.2% (44/271). Mortality was higher in patients with CMV pp65 antigenemia positivity than in those who were CMV pp65 antigenemia-negative (29% [24/82] vs 10.6% [20/189]). Of the 14 patients with CMV disease, 8 (57%) died, whereas 16 (24%) of the 68 patients with CMV infection died. Among the 24 patients who died, 20 received anti-CMV therapy (8 with CMV disease and 12 with CMV infection). The primary cause of death in both groups was infection, accounting for 5 deaths in the CMV disease group and 8 deaths in the CMV infection group (**Table 3**). Among infection-related deaths, sepsis/bacteremia was the most frequent cause in both groups (4/8 in CMV infection vs 4/5 in CMV disease), and the distribution of infection types did not show an apparent qualitative difference between groups (**Table 3**). The median time from treatment initiation to death was 136 days (interquartile range (IQR): 76–310) for CMV disease and 115 days (IQR: 56–169) for CMV infection.

**Table 3:** Details on the causes of death.

Cause of Death	CMV infection (n=16)	CMV disease (n=8)
Infection	8	5
Sepsis/bacteremia	4	4
Pneumocystis pneumonia	0	1
Invasive fungal infection	1	0
Bacterial pneumonia	3	0
Cerebral Infarction	0	1
Acute Exacerbation of Interstitial Pneumonia	1	1
Malignancy	3	0
Uremia	1	0
Unknown Cause	3	1
Total Deaths	16	8

CMV, cytomegalovirus.

**Figure 2:**
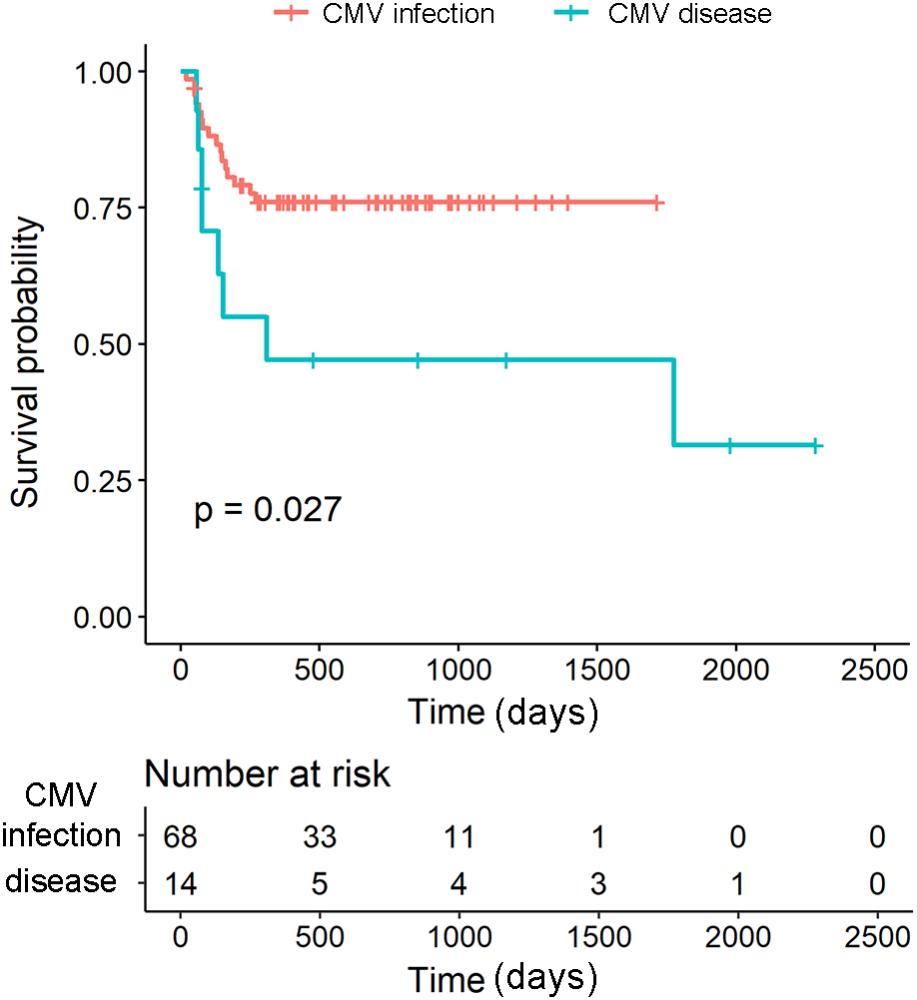
Survival probability of CMV disease and CMV infection. Kaplan–Meier analysis and the log-rank test demonstrated that patients with CMV disease had a significantly lower survival rate compared with those with CMV infection (log-rank test, p=0.027). The median time from the start of treatment to death for patients with CMV disease was 136 days (IQR: 76–310). CMV, Cytomegalovirus; IQR, Interquartile Range.

Kaplan–Meier analysis and the log-rank test revealed a significantly lower survival rate in patients with CMV disease than in those with CMV infection (log-rank, p=0.027) (**Figure 2**). Multivariate analysis using a Cox proportional hazards model showed that age ≥65 years had a hazard ratio (HR) of 3.2 (95% CI 1.07–9.37, p=0.027), and CMV disease had a HR of 2.6 (95% CI 1.05–6.26, p=0.039) (**Table 4**). These findings suggest that tissue-invasive CMV disease and old age may be associated with an increased risk of mortality.

**Table 4:** Cox proportional hazards model for mortality.

Variable	HR	95%CI	p value
CMV disease	2.6	1.05–6.26	**0.039**
≥ 65 years old	3.2	1.07–9.37	**0.027**

HR, hazard ratio; CI, confidence interval; CMV, cytomegalovirus.

Bold values indicate p-values less than 0.005.

## Discussion

This study is the first to investigate the characteristics of CMV disease during induction therapy for AIIRDs. In previous studies (5–8), the definition of CMV disease encompassed CMV syndrome with fever and cytopenia, treatment with antiviral drugs, and diagnosis by infectious disease specialists. In this study, we used a stricter definition of CMV disease, that is, CMV reactivation with tissue invasion. We observed that CMV disease was associated with higher antigen counts (cutoff value of 36), a high frequency of coincident infections, and a high mortality rate compared with CMV infection.

In the present study, 82 patients developed CMV pp65 antigenemia positivity within 3 months after initiation of remission induction therapy, corresponding to 30% among patients in whom CMV pp65 antigenemia was measured (82/271), which is broadly consistent with previous studies reporting CMV reactivation rates of approximately 18–30% in AIIRDs during induction therapy (6, 10–12). Notably, our cohort was older (median age 74 years) than those reported in prior studies (approximately 56–62 years) (6, 10–12). Although baseline CMV serostatus was not routinely available, CMV pp65 antigenemia positivity generally reflects CMV reactivation in previously infected individuals, and CMV seroprevalence is known to increase with age (13). Therefore, the older age profile of our cohort may have contributed to both the observed frequency of CMV pp65 antigenemia positivity and the high mortality. A previous study (8) has shown that the overall mortality rate within 3 months is higher in patients with organ damage due to CMV infection during treatment for systemic lupus erythematosus than in those without it. The mortality rate has also been reported to be higher in patients with CMV reactivation during treatment for AIIRDs than in those without reactivation (5, 10), but the mortality rate was not specifically related to CMV disease. In our study, we found that the mortality rate was higher in CMV disease than in CMV infection, indicating that the prognosis is worse for those with tissue damage than for those with reactivation alone. In particular, when patients aged ≥65 years present with clinical symptoms due to organ damage following CMV reactivation, a tissue biopsy should be taken for evaluation of CMV disease.

The present findings raise several points to explain the distinction between CMV disease and CMV infection. First, CMV reactivation occurs under immunosuppression, such as strong immunosuppressive therapy or high doses of glucocorticoids (12). In organ transplantation and AIIRDs, the dose of immunosuppressants, especially glucocorticoids, is considered a risk factor for CMV disease (12, 14), and stronger immunosuppression may increase the CMV viral load and cause CMV disease (1). Wu et al. (15) identified immunosuppression-related factors and higher viral burden as correlates of CMV infection. More recently, Ota et al. (16) prospectively monitored CMV pp65 antigenemia weekly in patients receiving remission induction therapy with high-dose glucocorticoids and reported that treatment regimen and comorbidity related factors were independently associated with CMV infection. However, in this study, CMV infection and CMV disease showed no differences in terms of steroid pulse therapy, maximum glucocorticoid dose, or type of concomitant immunosuppressant. Therefore, CMV reactivation associated with induction regimens may be less discriminative in this restricted population, and our study may have been underpowered to detect modest differences. CMV is an opportunistic pathogen, and previous studies have reported that CMV reactivation is associated with oral candidiasis and severe infections (5, 6). In this study, CMV disease was associated with coincident infections (OR 24.2). CMV suppresses the proliferation of activated T cells (17) and reduces the migration ability of macrophages (18), which may non-specifically weaken cellular and humoral immune responses and increase susceptibility to coincident infections (19). In this study, the maximum CMV antigenemia count was higher in CMV disease than in CMV infection, suggesting that patients with CMV disease may have higher CMV proliferation and stronger immunosuppression. Since CMV disease has a significantly higher mortality rate than CMV infection, active investigation of tissue-invasive CMV disease in the presence of opportunistic infections, such as oral candidiasis or coincident infections, is necessary.

Second, CMV disease may occur when the underlying disease has high severity. Severe inflammation is known to induce CMV reactivation (20–22). Inflammatory cytokines stimulate CMV replication, and CMV itself may activate cellular mechanisms that lead to further inflammation (19). In this study, the underlying disease was vasculitides in 52% of CMV disease cases, and thus more caution is required in inflammatory diseases.

CMV pp65-positive cell count >5.6 per 10^5^ polymorphonuclear leukocytes (7) and an average of five positive cells across two slides (C10/C11) (23) have been reported to be associated with CMV disease. In our study, the CMV antigenemia count predictive of CMV disease was 36, with a sensitivity of 79%, specificity of 84%, and AUC of 0.84. In organ transplantation, pre-emptive therapy and prophylactic administration of anti-CMV drugs are considered effective in preventing CMV disease (4, 24, 25). Pre-emptive therapy is often performed during remission induction therapy for AIIRDs, but no randomized controlled trials have compared the two approaches. Anti-CMV drugs, such as ganciclovir and foscarnet, are associated with adverse events, including cytopenia and nephrotoxicity (26). Pre-emptive therapy or early administration of anti-CMV agents when the CMV pp65-positive cell count is <5.6 per 10^5^ polymorphonuclear leukocytes or when there are fewer than five positive cells across two slides should only be considered if the benefits outweigh the risks. In some cases, it may be preferable to initiate pre-emptive therapy when the CMV antigenemia count is between 5 and 36.

This study has several limitations. First, it is a single-center, retrospective observational study with a small sample size. Second, the timing of CMV antigenemia measurement varied among cases, depending on the attending physician’s judgment. Therefore, the observed CMV positivity proportion and mortality difference are applicable only to patients in whom CMV pp65 antigenemia was measured and may not be generalizable to all patients receiving induction therapy. In addition, in patients for whom CMV antigenemia was measured later, CMV disease may have already developed. Third, since tissue invasion was not evaluated, CMV disease could have been underdiagnosed among cases classified in the CMV infection group. More severe cases of CMV disease may have been overrepresented in the CMV disease group. Fourth, the inclusion of different AIIRDs hindered accurate assessment of their severity and activity.

In summary, this study is the first to compare CMV disease, defined as evidence of CMV infection in organs damaged during induction therapy for rheumatic diseases, with CMV infection. CMV disease had a higher mortality rate and worse prognosis than CMV infection. Therefore, CMV disease should be actively screened when opportunistic infections, such as oral candidiasis or coincident infections, occur. In the future, multicenter evaluations and randomized controlled trials are needed to explore strategies for improving the management of CMV infection during the treatment of rheumatic diseases.

## Data Availability

The raw data supporting the conclusions of this article will be made available by the authors, without undue reservation.
