# Supplementary material for: Tissue-invasive CMV disease is associated with poor prognosis during remission induction therapy for autoimmune inflammatory rheumatic diseases
Source: Front Immunol. 2026 Feb 12;17:1696516. doi: 10.3389/fimmu.2026.1696516 (PMC12935964; doi:10.3389/fimmu.2026.1696516)
Supplement: Supplementary file 1 [file Table1.docx]

Supplementary Table 1

Types, timing, and severity of coincident infections relative to first CMV antigenemia positivity.

| Types of coincident infection | Before  first CMV antigenemia  positivity | On/after  first CMV antigenemia  positivity |
| --- | --- | --- |
| Oral and esophageal candida infections | 7 | 13 |
| Invasive pulmonary aspergillosis | 0 | 3 |
| Sepsis/bacteremia | 7 (1) | 10 (3) |
| Bacterial pneumonia | 1 | 5 |
| Urinary tract infection | 1 | 5 (1) |
| Cellulitis | 0 | 3 |
| Pneumocystis pneumonia | 0 | 2 (1) |
| Fungal infection | 0 | 1 |
| Pulmonary tuberculosis | 0 | 1 |
| Cholecystitis | 0 | 1 |
| Dental infection | 1 | 0 |
| Diverticulitis | 0 | 1 |

**Footnote.** Counts represent infection episodes that required initiation of antibacterial and/or antifungal therapy and occurred either before or on/after the first CMV antigenemia positivity. Patients could have more than one infection episode; therefore, counts may exceed the number of patients. Severe infection was defined as an infection requiring intensive care unit admission and/or vasopressor support. Numbers in parentheses indicate episodes classified as severe infections. Severe infections occurred in six patients (CMV infection, n=4; CMV disease, n=2).
